# Biohybrid Energy Storage Circuits Based on Electronically Functionalized Plant Roots

**DOI:** 10.1021/acsami.3c16861

**Published:** 2024-03-05

**Authors:** Daniela Parker, Abdul Manan Dar, Adam Armada-Moreira, Iwona Bernacka Wojcik, Rajat Rai, Daniele Mantione, Eleni Stavrinidou

**Affiliations:** † Laboratory of Organic Electronics, Department of Science and Technology, Linköping University, Norrköping SE-60174, Sweden; ‡ Neuronal Dynamics Laboratory, Department of Neurosciences, SISSA, International School for Advanced Studies, Trieste 34136, Italy; § POLYMAT University of the Basque Country UPV/EHU, Donostia-San Sebastian 20018, Spain; ∥ IKERBASQUE, Basque Foundation for Science, Bilbao 48009, Spain; ⊥ Wallenberg Wood Science Center, Linköping University, Norrköping SE-60174, Sweden; # Umea Plant Science Centre, Swedish University of Agricultural Sciences, Umea SE 90183, Sweden

**Keywords:** plants, biohybrid systems, supercapacitors, organic mixed ionic electronic conductors, in vivo polymerization

## Abstract

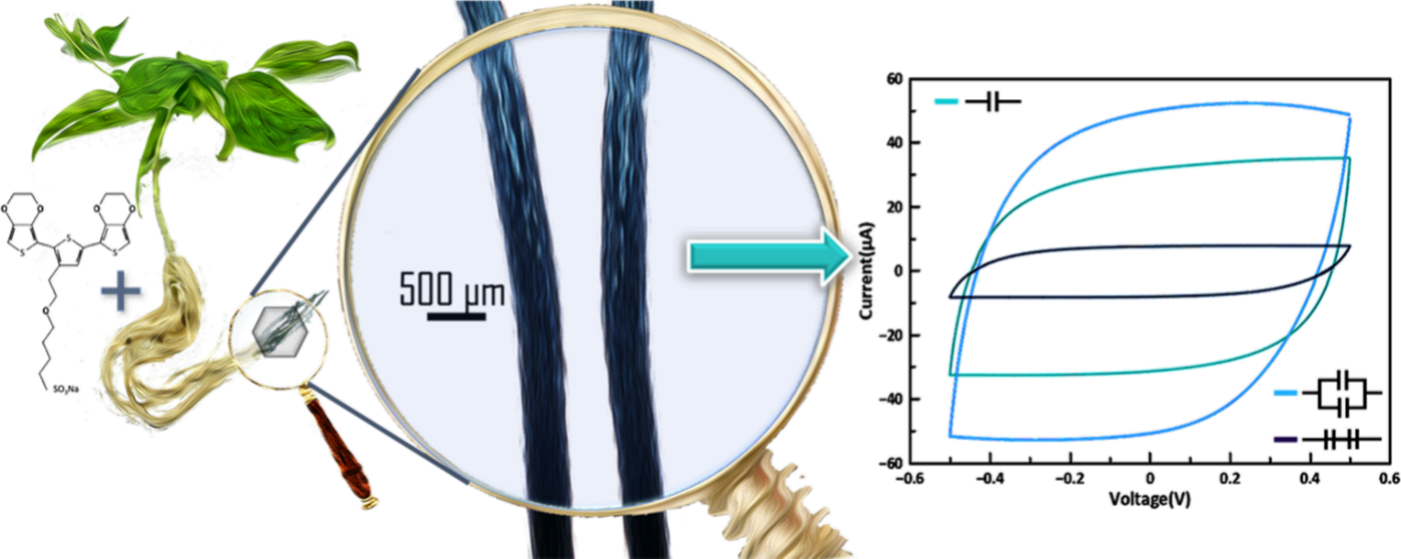

Biohybrid systems based on plants integrate plant structures and processes into technological components targeting more sustainable solutions. Plants’ biocatalytic machinery, for example, has been leveraged for the organization of electronic materials directly in the vasculature and roots of living plants, resulting in biohybrid electrochemical devices. Among other applications, energy storage devices were demonstrated where the charge storage electrodes were seamlessly integrated into the plant tissue. However, the capacitance and the voltage output of a single biohybrid supercapacitor are limited. Here, we developed biohybrid circuits based on functionalized conducting roots, extending the performance of plant based biohybrid energy storage systems. We show that root-supercapacitors can be combined in series and in parallel configuration, achieving up to 1.5 V voltage output or up to 11 mF capacitance, respectively. We further demonstrate that the supercapacitors circuit can be charged with an organic photovoltaic cell, and that the stored charge can be used to power an electrochromic display or a bioelectronic device. Furthermore, the functionalized roots degrade in composting similarly to native roots. The proof-of-concept demonstrations illustrate the potential of this technology to achieve more sustainable solutions for powering low consumption devices such as bioelectronics for agriculture or IoT applications.

## Introduction

Plant biohybrids are systems that are based on the amalgamation of plant structures and synthetic materials and/or devices resulting in systems with a hybrid functionality.^1−3^ Plants are autotroph organisms powered by the sun and at the same time convert carbon dioxide to oxygen. Biohybrid technologies that utilize plants natural processes and structures can therefore contribute to a more sustainable future. Furthermore, plants are particularly attractive for biohybrid systems as they are equipped with an active biocatalytic interface above and below the ground.^1,3^ They also sense and acclimate to their environment and exchange various molecules with it. In addition, plants have hierarchical structures that span many length scales and at the same time are carbon negative. Various approaches exist for developing plant biohybrids, either based on nanomaterials that target distinct plant organelles,^4^ materials that self-organize within plants extended tissues^5^ or devices that are integrated into plants.^6^

Tailormade nanomaterials that were incorporated directly in plants apparatus enabled the development of environmental sensors.^7,8^ The plant was acting as the sampler and concentrator of analytes while the nanomaterials produced a readable output. Functional nanomaterials can also enhance plant processes for example photosynthesis^8^ and CO_2_ uptake^9^ or even give new capabilities to plants such as light emission.^10,11^ Plants, being natural energy converters that transform sunlight to chemical energy, have also been the focus of biohybrid energy devices. Biofunctionalized electrodes integrated into the plant tissue can convert sugars and oxygen produced by photosynthesis into electricity.^12,13^ Additionally, plants’ natural movements and leaves’ dielectric properties have been combined with artificial leaves for triboelectric generators that produce sufficient energy to power low consumption electronics.^14,15^ Our group, on the other hand, demonstrated energy storage in plants via in vivo organization of conducting materials in their tissue that can act as charge storage electrodes in biohybrid supercapacitors.^16^

Utilizing plants’ biocatalytic activity to develop functional devices is indeed a promising avenue for biohybrid systems. We showed that plants could organize conjugated polymers and even polymerize conjugated trimers in vivo resulting in electrochemical devices that are seamlessly integrated into the plant structure.^17,5^ Specifically, we discovered that peroxidase enzymes that are present in the plants cell wall could polymerize the conjugated trimer 4-[2-{2,5-bis(2,3-dihydrothieno[3,4-*b*][1,4]dioxin-5-yl)thiophen-3-yl}ethoxy]butane-1-sulfonate sodium salt, ETE-S, with the conducting polymer being integrated along the plant cell wall.^18,19^ The integrated conducting wires in the plant’s vasculature had conductivity of 10 S/cm and specific capacitance of 20 F/g.^17^ While initial work focused on plant cuttings, recently we developed intact biohybrid plants with an electronic root system.^16^ The integrated mixed ionic–electronic conductors in the roots maintained their functionality over weeks and as a proof of concept we demonstrated energy storage by forming a root-based supercapacitor. However, the voltage output of a single root supercapacitor is limited to less than 1 V, and the capacitance to few mF, therefore restricting potential applications.

Apart from biohybrid approaches, materials from plants have been used as structural components for supercapacitors. Various forms of cellulose, the main component of the plant cell wall and the most abundant biopolymer on Earth, have been combined with carbon-based materials and/or conjugated polymers to form charge storage electrodes, via coating, blending or in situ polymerization.^20−22^ PEDOT a thiophene derivative polymer has been widely explored in combination with cellulose due to its high electronic and ionic conductivity, volumetric capacitance but also its solution processability, enabling the development of paper like supercapacitors that can be fabricated with printing technology, opening the possibility for low cost and large-scale production.^23−25^ In addition to cellulose, wood has also been explored for supercapacitors, as its inherent porosity results is high surface area that is advantageous for charge storage.^26^ Wood-based charge storage electrodes were fabricated by carbonization and/or further functionalized with conjugated polymers and metal oxides.^27,28^ While approaches based on extracted plant materials can result in high performing devices, devices based on living plant tissue can be seamlessly integrated within the living organism, offering the advantage of harnessing the endogenous biochemical environment for device fabrication.

In this work, we extended the concept of energy storage in plants and developed biohybrid circuits based on ETE-S functionalized conducting roots. We demonstrate that p(ETE-S) roots can be used as charge storage electrodes to form supercapacitors that are stable over 500 cycles, with 98% Coulombic efficiency and with an average capacitance of 3.6 mF. By connecting the root-supercapacitors in series and parallel configuration, we extended the voltage and current range. As a proof of concept, we charged the biohybrid circuit with an organic photovoltaic cell and then delivered the charge to power an electrochromic display and a bioelectronic device. Finally, we tested the degradability of the functionalized roots in soil with compost activator.

## Results and Discussion

**Figure 1:**
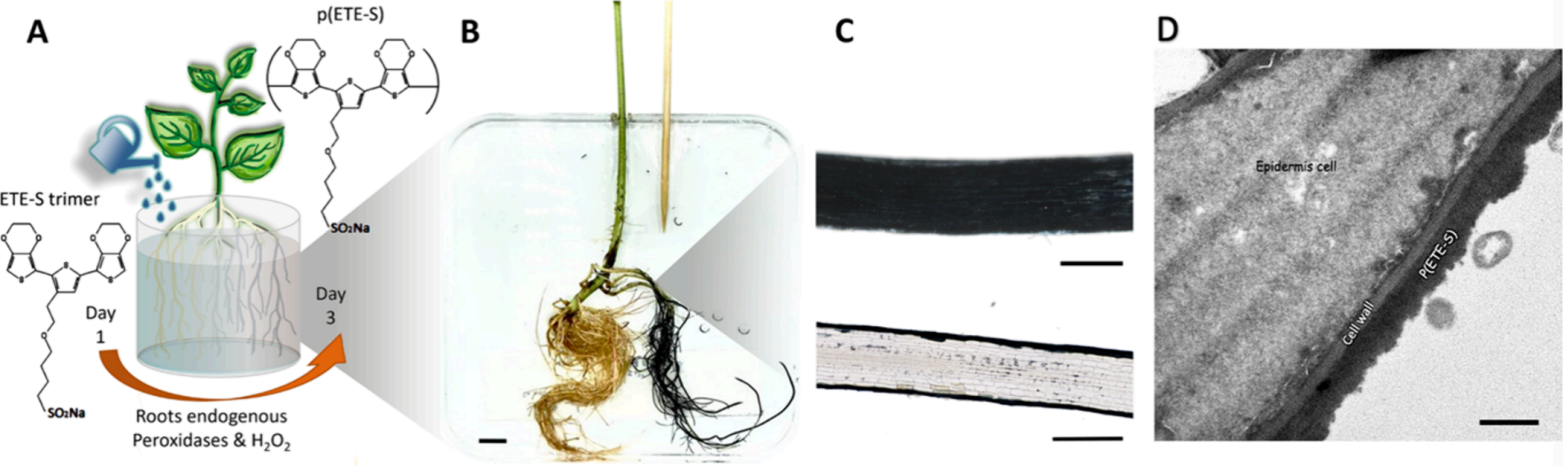
Functionalization of bean roots with p(ETE-S). (A) Schematic of bean roots functionalization with ETE-S trimer. (B) Photograph of bean plant root system with p(ETE-S) roots on the right (dark roots), scale bar 1 cm. (C) p(ETE-S) fixed root micrographs in plane view and longitudinal cross section, scale bar 500 μm. (D) TEM micrograph of p(ETE-S) root cross section, scale bar 1 μm.

The roots of bean plants were electronically functionalized via in vivo polymerization of ETE-S, as described previously.^16^ Selected roots that were still attached to the plant were immersed in individual containers with 1 mg/mL ETE-S solution for 72 h (Figure 1A, B). The p(ETE-S) roots were detached from the plant after functionalization for characterization and device fabrication. As studied in detail in our previous work and here shown in Figure 1C, ETE-S polymerizes to p(ETE-S) forming a few micrometer thick homogeneous layer on the epidermis–exodermis of the root.^16^ Wide angle X-rays scattering also previously revealed the π–π stacking of the thiophene rings suggesting the favorable organization of the p(ETE-S) chains along the root.^16^ To get further insight on the layer formation, here we preformed Transmission Electron Microscopy and show that the p(ETE-S) layer is homogeneous and adheres very well to the cell wall (Figure 1D).

**Figure 2:**
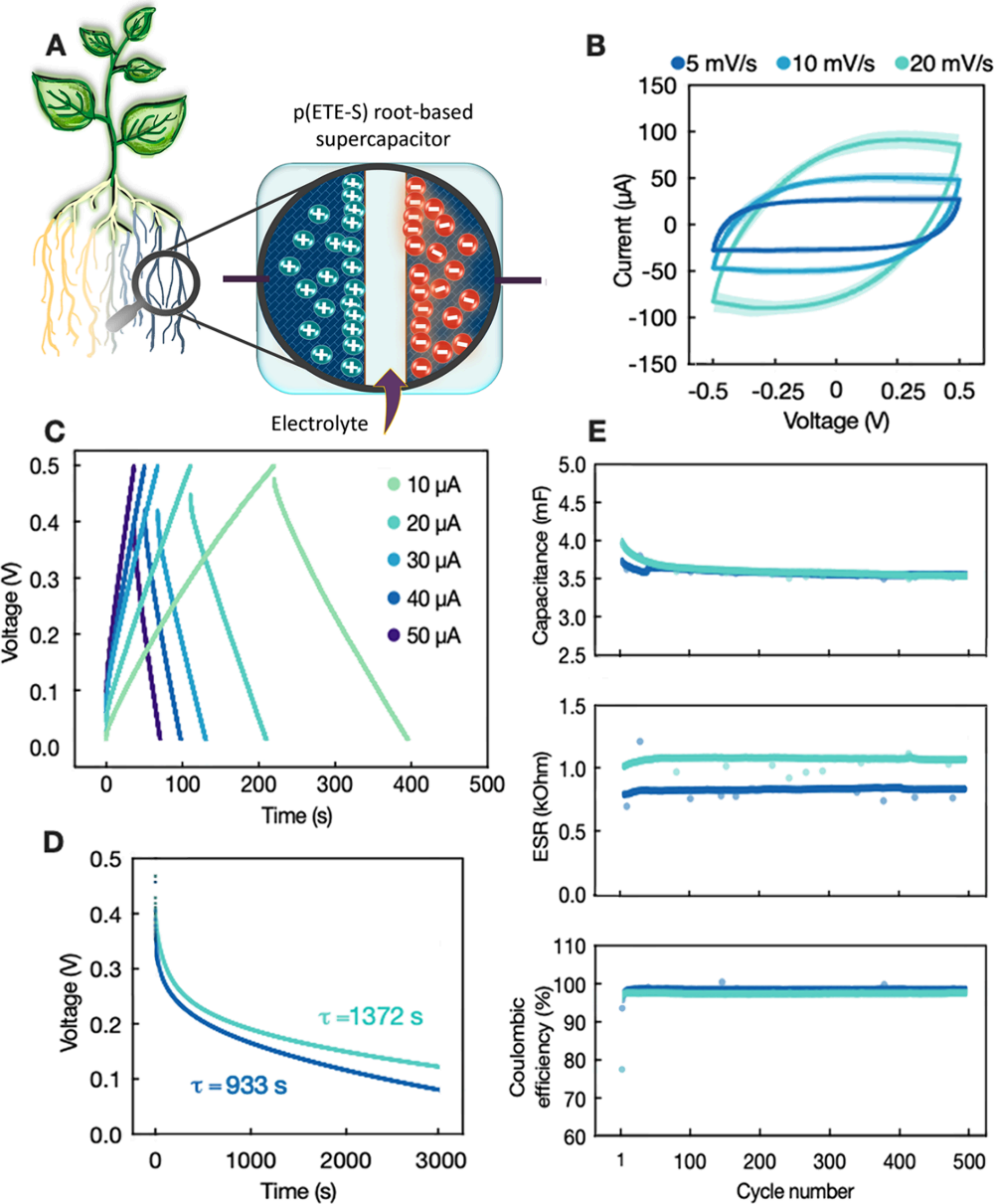
Characterization of p(ETE-S) root-based supercapacitor. (A) Schematic of bean roots based supercapacitor. (B) Average cyclic voltammogram of p(ETE-S) root-based supercapacitor at 5, 10, and 20 mV/s scan rate, with halo as standard deviation, *n* = 4 (first, 4 to 5 CV cycles of each supercapacitor were averaged; then those were averaged by scan rate). (C) Typical galvanostatic charge–discharge curves for applied currents of 10–50 μA and *V*_max_ = 0.5 V. (D) Capacitance, equivalent series resistance and Coulombic efficiency over galvanostatic cycling for *I* = 30 μA and *V*_max_ = 0.5 V, *n* = 2. (E) Self-discharge characteristics, *n* = 2.

To form a supercapacitor, two electronic roots were arranged in parallel with the help of a 3D printed holder separated by 0.01 M KCl electrolyte (Figure 2). The roots were addressed with carbon fiber/carbon paste -based electrodes. Cyclic voltammetry revealed capacitive charging, as indicated by the box shape of the graph and the current plateau that scales linearly with scan rates up to 20 mV/s (Figure 2B). The electrochemical response agrees with the current understanding of p(ETE-S) mixed ionic-electronic conduction properties. The p(ETE) backbone carries positive electronic carriers while the sulfonate groups on the side chains facilitate ion transport in the polymer bulk. During charging, positive charges are injected into the polymer backbone that are stabilized by the sulfonate groups and by other anions that enter from the electrolyte. During discharge, positive carriers are extracted from the backbone while mobile dopants are expelled from the polymer film and the sulfonate groups are compensated by mobile cations. The capacitance was calculated from galvanostatic charging–discharging curves and was found to be 4.8 mF for charging current of 10 μA, while it decreased to 3.9 mF for 50 μA (Figure 2C, Table S1). The equivalent series resistance (ESR) that accounts for the resistive components of the circuit was equal to 1.41 and 1.36 KΩ for 10 μA and 50 μA, respectively. Overall, we observed very good device to device reproducibility, as four supercapacitors with different sets of p(ETE-S) roots showed very similar behavior (Figure S1), also in agreement with our previous published work.^16^ Furthermore, the root electrochemical properties were not affected by their storage time (hydrated, in the fridge), as supercapacitors from roots with different storage time performed similarly (Figure S2). Indeed, we have previously shown that the p(ETE-S) roots maintain their conductivity for at least 4 weeks even if the roots are still attached on a growing plant.^16^ We then characterized the cycling stability and self-discharge behavior. We found that the root-supercapacitors were stable for over 500 charge–discharge cycles with Coulombic efficiency (*Q*_charging_/*Q*_discharging_) close to 100% (Figure 2D). The average capacitance over cycling was 3.6 mF, while the ESR was 956 Ω (Figure 2D). By charging the device and then monitoring the open circuit potential, we found that the root-supercapacitor discharged after 20 min (Figure 2E).

**Figure 3:**
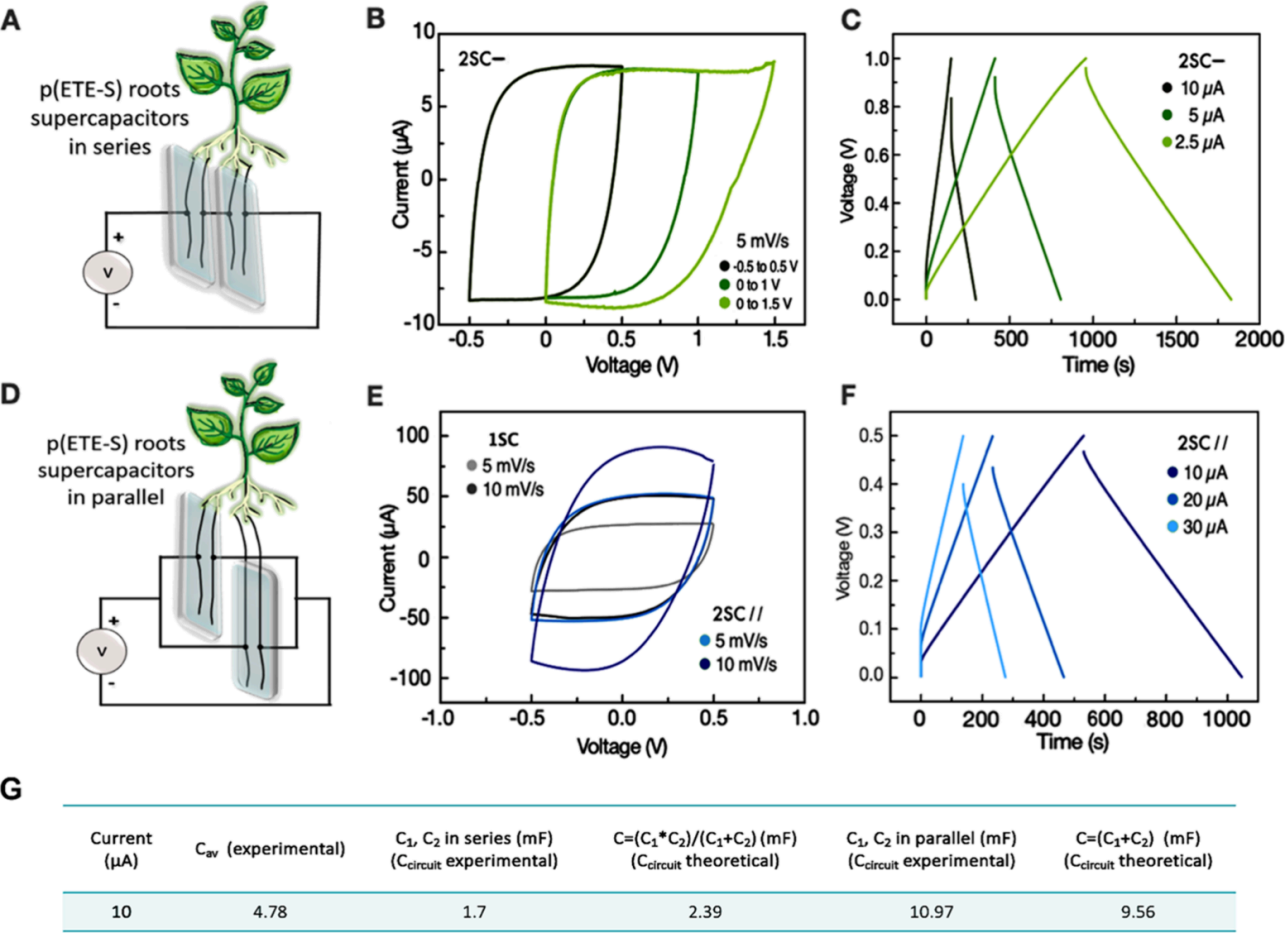
Characterization of two p(ETE-S) root-based supercapacitors in series and in parallel connection. (A) Schematic of supercapacitors in series. (B) Cyclic voltammogram of supercapacitors in series at 5 mV/s scan rate; from −0.5 to 0.5 V, 0 to 1 V, and 0 to 1.5 V. (C) Galvanostatic charge–discharge curves of supercapacitors in series with applied current 2.5–10 μA and *V*_max_ = 1 V. (D) Schematic of supercapacitors in parallel. (E) Cyclic voltammogram of a single supercapacitor and two supercapacitors connected in parallel at 5 and 10 mV/s scan rate. (F) Galvanostatic charge–discharge curves of two supercapacitors in parallel with applied current 10–30 μA and *V*_max_ = 0.5 V. (G) Experimental values and theoretical calculations of total capacitance for in series and in a parallel circuit.

To investigate whether it is possible to increase the performance of the biohybrid energy storage devices, we combined several roots to form biohybrid circuits of supercapacitors in series and in parallel. When two p(ETE-S) root supercapacitors were connected in series, the operating voltage of the circuit extended from 0.5 to 1.5 V (Figure 3A–C). At the same time, the capacitance at 10 μA decreased from 4.8 mF to 1.7 mF. In contrast, when we connected two supercapacitors in parallel the capacitance increased to 11 mF (Figure 3D, E, F). While the voltage range remains the same as in the single supercapacitor, in the parallel connection configuration, the charging current doubles as seen from the CVs (Figure 3E). Considering the average capacitance value at 10 μA, as measured for the single supercapacitors, we calculated the theoretical value of the total capacitance of the series and parallel circuit (Figure 3G). The theoretical value of the total capacitance for the parallel and series configuration was 9.56 mF and 2.39 mF, respectively, while the measured values were 10.97 mF and 1.7 mF, signifying that the biohybrid circuits follow the classical circuit analysis. However, the circuits are not ideal, and there is a difference between experimental and theoretical value (Δ*C*% = [(*C*_exp_ – *C*_th_)/*C*_th_]100) of 29% for the series circuit and 15% for the parallel circuit (Figure 3G).

**Figure 4:**
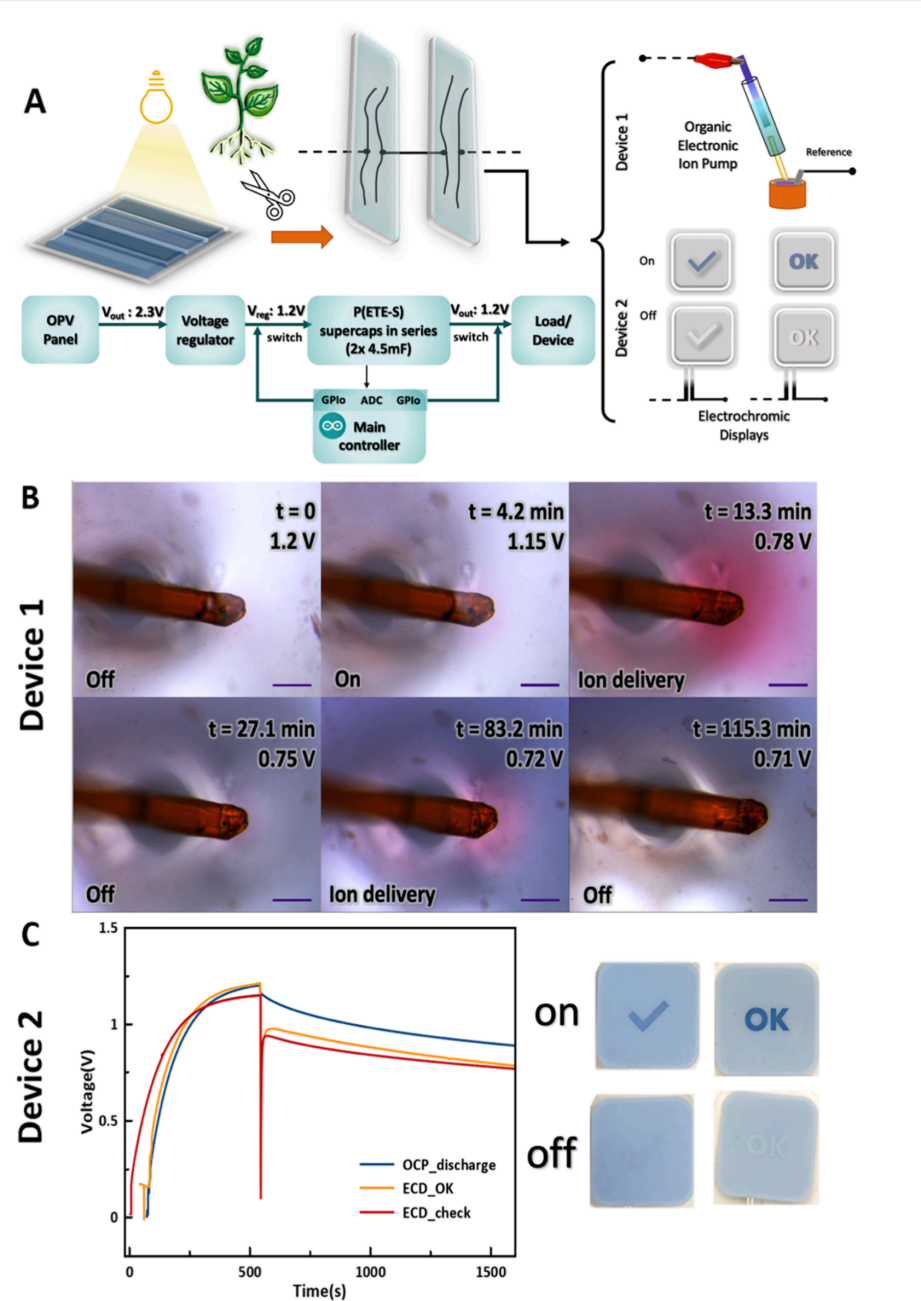
Root supercapacitor circuits demonstrators for powering devices. (A) Two p(ETE-S) root-based supercapacitors connected in series are charged by an OPV cell up to 1.2 V. The stored charges are used to power an OEIP or ECD. (B) Micrographs of the pH indicator solution during operation of the OEIP powered by the supercapacitor circuit. The proton delivery triggered color changes of the pH indicator from yellow to red. *t* indicates the time passed from the onset of charge delivery to the device via the supercapacitor circuit and the voltage across it. Scale bar 100 μm. (C) Voltage across the supercapacitor circuit over time while being charged by the OPV and then in open circuit or when the charged is delivered to the ECDs.

We then explored the functionality of the root-supercapacitors to demonstrate their ability to store energy and power devices in more complex circuits (Figure 4). Supercapacitors in series were charged by a commercial organic photovoltaic cell (OPV, Epishine) and were then used to power an electrochromic display (ECD) or a bioelectronic device. The OPV delivers up to 2.5 V, depending on the intensity of the light, while two supercapacitors in series can be charged up to 1.5 V. To reduce the output voltage of the OPV, we added a voltage divider circuit comprised of two resistors of 100 kΩ, resulting in output voltage of 1.2 V. An Arduino microcontroller was used to read the voltage, and as soon as the output voltage of the supercapacitors circuit reached 1.2 V, the microcontroller sent a control signal to activate a relay to switch from charging to discharging and to power the connected device, either the ECD or the bioelectronic device.

In the first demonstration, we choose to power a bioelectronic device, the organic electronic ion pump (OEIP). The OEIP is an electrophoretic delivery device that is used for controlled delivery of ions or charged biomolecules.^29^ Capillary-based OEIPs typically operate with applied voltage between 0.5 V-1.5 V and have been also used for in vivo hormone delivery in plants.^30,31^ To demonstrate the operation of the OEIP powered by the biohybrid circuit, we delivered protons in an electrolyte containing a pH indicator (Figure S3). When protons were delivered from the device outlet in the solution, the color of the solution changed from yellow to red due to the decrease in pH (Figure 4B). A few minutes after delivery, the color of the solution changed, while at the same time the voltage across the supercapacitors decreased to 0.78 V. Within 2 h, the OEIP was turned off and on several times and the voltage across the energy storage unit hold to around 0.7 V (Figure 4B, Video S1). For the second demonstrator we used the energy stored in the supercapacitors to power two PEDOT:PSS (poly(3,4-ethylenedioxythiophene): polystyrenesulfonate) based electrochromic displays (RISE), with different active display pattern. We observed that the ECDs turned on within 10 s and that they consumed similar charge as shown by the output voltage of the supercapacitors (Figure 4C). We also tested the self-discharge of the supercapacitors circuit. When we disconnected the OPV from the demonstrator after charging the supercapacitors to 1.2 V, the voltage across the circuit halved after 1.6 h. However, if all the circuitry was disconnected, the voltage across the supercapacitor circuit remained above 0.6 V for 2.5 h (Figure S4).

**Figure 5:**
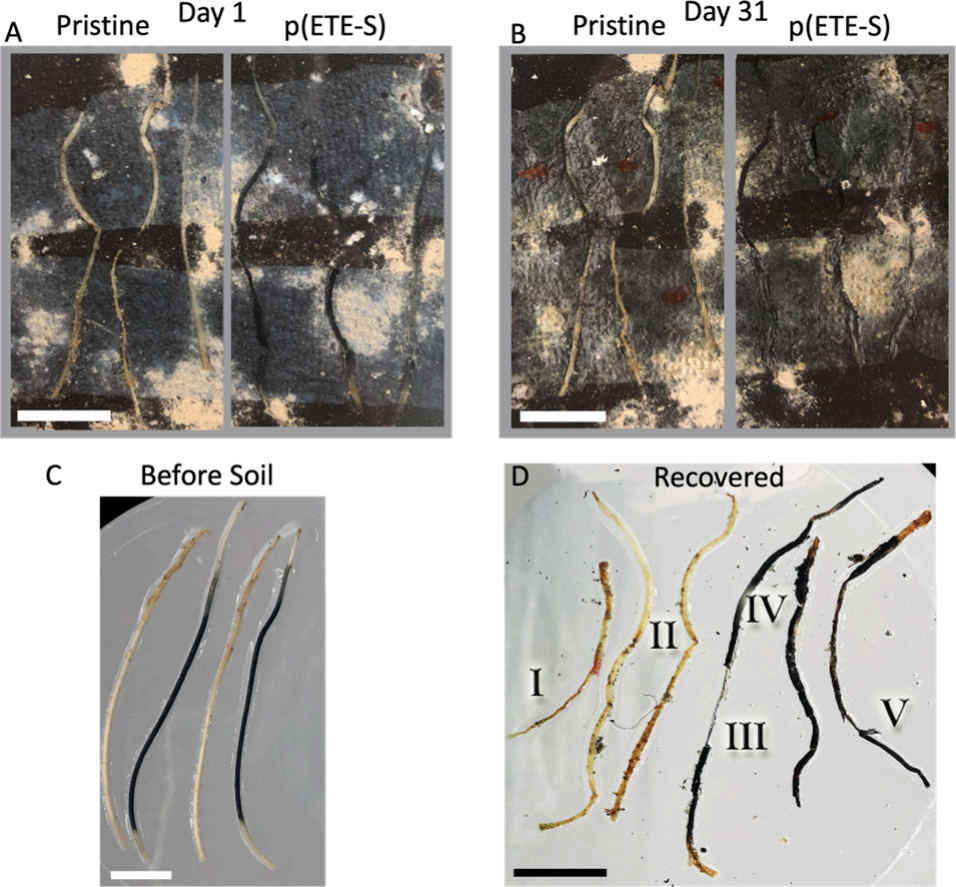
Degradability of the p(ETE-S) roots. (A) Day 1 and (B) Day 31 of the degradability test with the nonfunctionalized roots (pristine) on the left and the p(ETE-S) roots on the right. (C) Typical view of pristine and p(ETE-S) roots after they are harvested from the plant and prior degradation test. (D) The roots (of A and B) were recovered after day 31 and they show similar degradability. We noted areas of *I*–*V* as examples of degradation effect: (I) the pristine root has shrunk; (II) fractured areas of pristine roots; (III) in p(ETE-S) root, the main part of the root is missing; (IV) fractured p(ETE-S) root; (V) tissue delamination in p(ETE-S) root. Scale bar, 1 cm.

Finally, we qualitatively evaluated the degradability of the p(ETE-S) roots. p(ETE-S) roots and pristine - nonfunctionalized roots were placed together (*n* = 3 for each case) in a sealed glass Petri dish with moist soil and a mix of compost accelerator (Figure 5). The roots were fixed at the bottom of the container so that we could monitor their degradability over time. We took micrographs of marked zones every week for a month (Figure S5). Afterward, we recovered the roots and we observed that both functionalized and nonfunctionalized roots degraded to a similar extent (Figure 5D). For comparison, we show how pristine, and p(ETE-S) roots look like just after their harvest from the plant (Figure 5C). However, when establishing the biodegradation assay, some wounding and bending may occur in the roots from the handling. While this is a qualitative assessment, in the degraded roots, we marked some areas where the degradation effect is more obvious; for example, the root has shrunk or is fractured or tissue is missing.

## Conclusions

By harnessing the structure and the biocatalytic machinery of plants root system, we demonstrate a simple way of fabricating charge storage electrodes just by immersing plant roots in the ETE-S solution. The p(ETE-S) root supercapacitors are stable over cycling and can be connected in series or in parallel.

We showed that by connecting two supercapacitors in series, we can extend the voltage output to 1.5 V, while in parallel connection, we can achieve capacitance of 11 mF. In the future, even more complex circuits can be achieved by a combination of in series and in parallel connections, therefore extending both voltage output and capacitance. The p(ETE-S) root supercapacitor has energy density of 0.55 Wh/kg and power density of 150 W/kg. The energy density is comparable with conventional PEDOT:PSS-cellulose based supercapacitors fabricated with spray coating on flexible substrates.^32^ However, the power density is 2 orders of magnitude lower^32^ due to the high ESR of the root supercapacitors. While the in vivo polymerized p(ETE-S) has specific capacitance of 20 F/g^5^ that is comparable to PEDOT based materials,^23^ the use of a point contact on the p(ETE-S) root as a current collector results in large ESR values (kΩ range). Notably, no other processing is done on the p(ETE-S) layer to enhance its electroactive properties after it is formed via the in vivo polymerization, while in conventional devices, various additives are used to improve the charging of the electroactive material. The roots supercapacitors are also comparable in terms of stability and Coulombic efficiency with the aforementioned PEDOT:PSS-cellulose devices.

As a proof of concept, we developed demonstrators where the biohybrid circuit is used to power low consumption devices. The circuit is charged by a commercial organic photovoltaic cell specific for indoor energy harvesting. In the first demo, we powered an organic electronic ion pump that has been used for targeted hormone delivery in plants,^30,31^ thus showing the potential of the circuit for powering bioelectronic devices for agriculture. In the second demo, we chose to power an electrochromic display, illustrating the possibility for using such circuits for the Internet of Things (IoT) such as smart labels. To form the supercapacitors and circuits, we used detached roots mostly to facilitate the experiments, as the current method for electrical addressing of the roots requires the use of a fixed set up. Our methodology though opens the pathway for fabricating charge storage electrodes using the natural plant tissue as a substrate and the plants’ biochemical processes for the formation of the active layers. With increasing demands for sustainable energy storage devices, we further show that the functionalized roots degrade similarly to nonfunctionalized roots and can be a promising solution for low energy consumption devices. On the other side, future work may focus on developing flexible connectors that adhere to the root, enabling the operation of these circuits while still being part of the plant. As shown in our previous work, the functionalized roots that are attached to the plant maintain their electronic properties for at least 4 weeks while the plant continues its growth and development.^16^ Another limitation of the biohybrid circuit is the self-discharge that does not enable long-term energy storage. Nonetheless, by combining the supercapacitor circuit with an energy harvesting device, the accumulated charge can be sufficient for powering devices. In this work we used a commercially available OPV to charge the supercapacitors, but by using a plant-based energy harvesting device such as a biofuel cell or a triboelectric generator, a plant based autonomous energy system can be realized. For example, such systems can be used in agriculture or for environmental monitoring or seamlessly integrated in the urban living space for the IoT.

## Materials and Methods

### Plant Growth

Seeds of *Phaseolus vulgaris* collected from plants grown in our greenhouse were germinated in commercial plant starter scaffolds (Root Riot). The scaffolds were first moistened with deionized water and the seeds placed in the middle. Each scaffold was fixed in a transparent cone, sealed with parafilm, and incubated in complete darkness at 20–22 °C for five to seven days. After seed germination, the scaffold was removed from the seedlings, and the seedlings were put in containers filled with nutrient solution (0.5% v/v in tap water, Hyponex) and placed in the greenhouse under the following growth conditions: temperature 23–27 °C, humidity 50–70%, with a cycle of 12 h daylight with light intensity in the range of 80–100 μmol m^–2^ s^–1^.

### ETE-S Synthesis

ETE-S trimer, 4-[2-{2,5-bis(2,3-dihydrothieno[3,4-*b*][1,4]dioxin-5-yl)thiophen-3-yl}ethoxy]butane-1-sulfonate sodium salt, was synthesized as previously.^18^ Briefly, 3-thiopheneethanol has been 2,5-dibrominated with N-bromosuccinimide, then a Suzuki reaction catalyzed with PdPEPPSI using two equivalents of a 2-borolane-EDOT derivative give the hydroxy terminated-ETE derivate. To self-dope the trimer, a ring-opening allows the attachment of the sulfonate derivative, using buthane sultone.

### Plant Root Functionalization with ETE-S

Roots from 4 to 6-week-old plants while still attached to the plant were selected and washed 2 times with tap water. Then they were immersed in 1 mg/mL of freshly prepared ETE-S aqueous solution within a sealed pipet tip for 72 h. During functionalization the rest of the root system was immersed in a nutrient solution (0.5% v/v in tap water, Hyponex). After functionalization, treated roots were removed from the pipet tips and cut from the plant. The functionalized roots were stored individually in pipet tips filled with tap water and placed in a closed Petri dish at 4 °C until further characterization.

### p(ETE-S) Root Supercapacitor Fabrication and Characterization

The charge storage electrodes of a supercapacitor were defined by two p(ETE-S) roots. The p(ETE-S) roots were fixed on a 3D printed setup that had a defined separation between the two roots of 5 mm, and 0.01 M KCl was used as the electrolyte. The p(ETE-S) roots were contacted on the surface by a bundle of carbon fibers fixed with carbon paste (fibers bundle, ∼500 μm thick). The carbon fibers were addressed with probes tips (Au-plated tungsten tip of 50 μm diameter, coated with carbon paste) with the help of micromanipulators.

The individual supercapacitors and the configuration in series and parallel were characterized using cyclic voltammetry and galvanostatic charging–discharging using a Keithley K2600B source measure unit controlled by a custom-made Lab-view program. For the cyclic voltammogram one of the roots served as a working electrode, while the other one as counter/reference electrode. The supercapacitors in the galvanostatic measurements were switched from charging to discharging as soon as the set maximum voltage was reached. We used a simple equivalent *R*, *C* circuit in series to fit the discharging response of the supercapacitor. The resistor represents the equivalent series resistance.

The supercapacitor capacitance *C* and the ESR were calculated using the following relations:
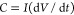
where *I* represents the applied current and d*V* /d*t* represents the slope of the discharging curve as done in standardized characterization of supercapacitors (IEC 62576).
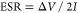
where *I* represents the applied current and Δ*V* represents the voltage drop at the beginning of the discharging curve.

The supercapacitor Energy Density and Power Density were calculated using the following relations:
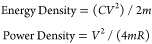
where *C* is the capacitance, *V* is the operating voltage, *R* is the ESR, and *m* the mass of the electroactive material in one electrode.

### OEIP Fabrication

The OEIP fabrication was adapted from the protocol described in ref (30); however, in the current work instead of anion-exchange membrane, a cation-exchange membrane 2-acrylamido-2-methyl-1-propanesulfonic acid sodium salt (AMPS) was applied in the glass capillaries of larger hollow (50 μm ID). Shortly, the 45 cm long sections of polyimide-coated fused silica capillaries (ID 50 μm; OD 150 μm, TSP050150, Polymicro Technologies LLC, UK) were connected to a nitrogen supply line to give the flow rate of 0.5 bar. The capillaries were flushed with 2 M KOH for 2 h, then with DI water for 10 min and dried by N_2_ flushing for 10 min. In the following step, 3-(trimethoxysilyl)propyl methacrylate (10 wt % in toluene) was flushed for 1 h, followed by ethanol flushing for 20 min and drying through N_2_ flushing for 10 min. Finally, the capillaries were filled with the polyelectrolyte mix containing monomer AMPS (50 wt %% in DI water, Sigma-Aldrich), cross-linker Poly(ethylene glycol) diacrylate (PEGDA, molecular weight 575 g/mol, 2 wt %, Sigma-Aldrich), the two photoinitiators: 2-Hydroxy-4′-(2-hydroxyethoxy)-2-methylpropiophenone (Igracure 2959; 0.5 wt %, Sigma-Aldrich) and Lithium phenyl-2,4,6-trimethylbenzoylphosphinate (LAP, 0.5 wt %, Sigma-Aldrich). The monomers in the capillaries were photopolymerized for 170 min using a homemade photoexposure box with four blue light lamps DULUX L BLUE 18 (Osram, Sweden). The OEIP capillaries were then stored in NaCl 0.1 M solution until use. In the device assembling process, the capillaries were cleaved into ∼1 cm long sections that were individually attached to polyolefin heat shrink tubing (5–6 cm long), which serve as the reservoir for the source electrolyte.

### OEIP-Mediated H^+^ Delivery

The OEIP reservoir was filled with aqueous 0.1 M HCl solution. The delivery tip was immersed in the target electrolyte that consisted of the pH indicator methyl red (2.5 mM; Sigma-Aldrich) and 0.01 M KCl (Sigma-Aldrich). The OEIP was loaded with H^+^ ions by applying constant current of 750 nA using Keithley 2602 source-meter, using two electrodes made of a poly(3,4-ethylenedioxythiophene):polystyrenesulfonate (PEDOT:PSS). Once the device was loaded, the roots supercapacitors were attached to supply the voltage for the OEIP-mediated H^+^ delivery. The H^+^ delivery triggered color changes of the pH indicator from yellow to red at pH below 6.2. The color changes were visualized and analyzed using stereomicroscope (Nikon, Sweden).

### Demos: OPV-SCs-ECD/OEIP

For charging the supercapacitors array (either in series or parallel), an organic photovoltaic cell was utilized with a voltage divider circuit of two 100 kΩ resistors to down regulate its output voltage from 2.3 V (at indoor illumination of 11 μ mol/m.^2^s^1^), to 1.2 V, as an input voltage to supercapacitors. An Arduino Uno microcontroller controlled the charging and discharging of the root supercapacitor by reading the potential via the Analog to digital converter (ADC, 12 bit) input and sending a control signal to the relay module (VMA406, Velleman) that controlled the switching between the charging of OPV-supercapacitors array and discharging to the Load (ECD or OEIP). During charging the OPV is electrically connected through a switch to charge the root supercapacitor and disconnects automatically after reaching the final voltage of 1.2 V. Right after the switch disconnects the OPV cell, the discharging of the p(ETE-S) root-based supercapacitor array, powers the connected electronic load.

### Degradation Assay

Nonfunctionalized and p(ETE-S) functionalized bean roots were placed in a glass Petri dish and fixed with wet strips of tissue. First, they were sprinkled with 4 mg of commercial compost accelerator (Green Line, Dolomite, Urea, (NH_4_)_3_PO_4_, K_2_SO_4_, Guano) and then a layer of 1 cm of commercial soil was added, mixed with vermiculite to avoid soil compression while adding water. The soil was moistened with 15 mL of tap water. On top of the soil, a layer of 5 mm of commercial swelling gel (Swell Gel) was added to prevent the soil from drying during the whole duration of the experiment. Finally, the Petri dish was closed and sealed with parafilm. The Petri dish was placed in a dark environment at room temperature.

## Supporting Information Available

Device to device reproducibility, stability of functionalized roots, details on proof-of-concept demonstrators and degradability assay (PDF)
