# Supplementary material for: Biohybrid Energy Storage Circuits Based on Electronically Functionalized Plant Roots
Source: ACS Appl Mater Interfaces. 2024 Mar 5;16(45):61475–83. doi: 10.1021/acsami.3c16861 (PMC11565472; doi:10.1021/acsami.3c16861)
Supplement: Supplementary file 2 — am3c16861_si_002.pdf [file am3c16861_si_002.pdf]

Supporting Information

Biohybrid energy storage circuits based on electronically functionalized plant roots

Daniela Parker[a], Abdul Manan Dar[a], Adam Armada-Moreira[a,b], Iwona Bernacka Wojcik[a], Rajat Rai,[c] Daniele Mantione[c,d], Eleni Stavrinidou[a,e,f,*]

a Laboratory of Organic Electronics, Department of Science and Technology, Linköping University, SE-60174, Norrköping, Sweden

b Neuronal Dynamics Laboratory, Department of Neurosciences, SISSA, International School for Advanced Studies, 34136 Trieste, Italy

c POLYMAT University of the Basque Country UPV/EHU, 20018 Donostia-San Sebastian, Spain.

d IKERBASQUE, Basque Foundation for Science, 48009, Bilbao, Spain.

e Wallenberg Wood Science Center, Linköping University, SE-60174, Norrköping, Sweden

f Umea Plant Science Centre, Swedish University of Agricultural Sciences, SE 90183 Umea, Sweden.

*Corresponding Author: eleni.stavrinidou@liu.se

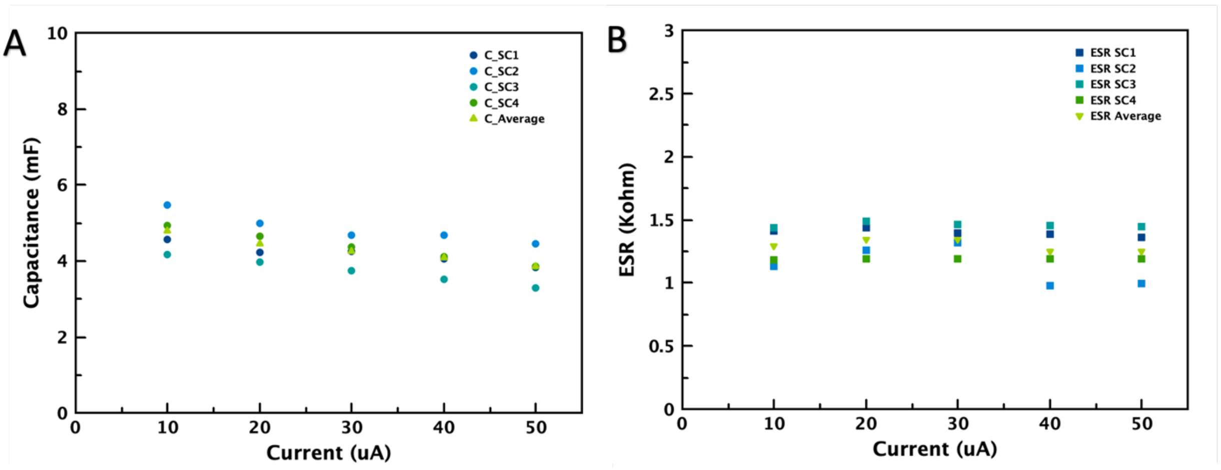

Figure S1. (A) Capacitance and (B) Equivalent series resistance of four different p(ETE-S) root-based supercapacitors (SC1-4), and their average capacitance for different charging currents.

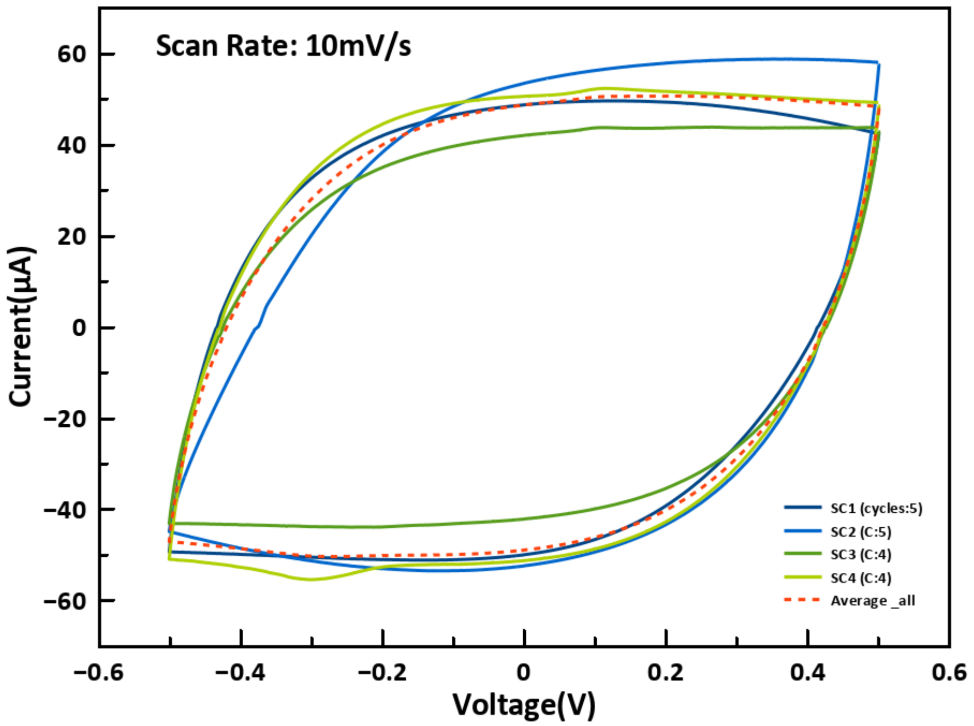

Figure S2. Average cyclic voltagramm of four supercapacitors at 10 mV/s scan rate (red dashed line). Each curve represents the average CV of a different supercapacitor (average of 4 to 5 cycles). SC1 and SC2 correspond to roots that stayed less time in the fridge (2 weeks), while SC3 and SC4 correspond to roots staying in the fridge for longer period (4 weeks).

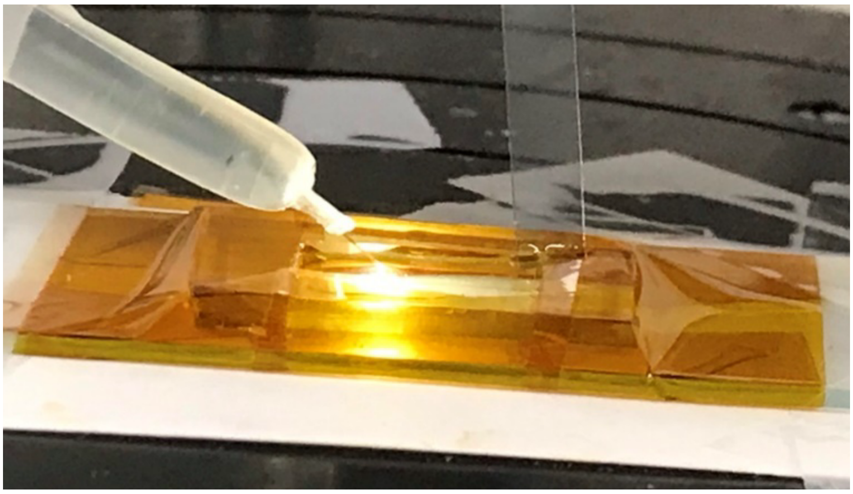

Figure S3. Setup for OEIP-mediated H+ delivery under the microscope, powered by the supercapacitor circuit.

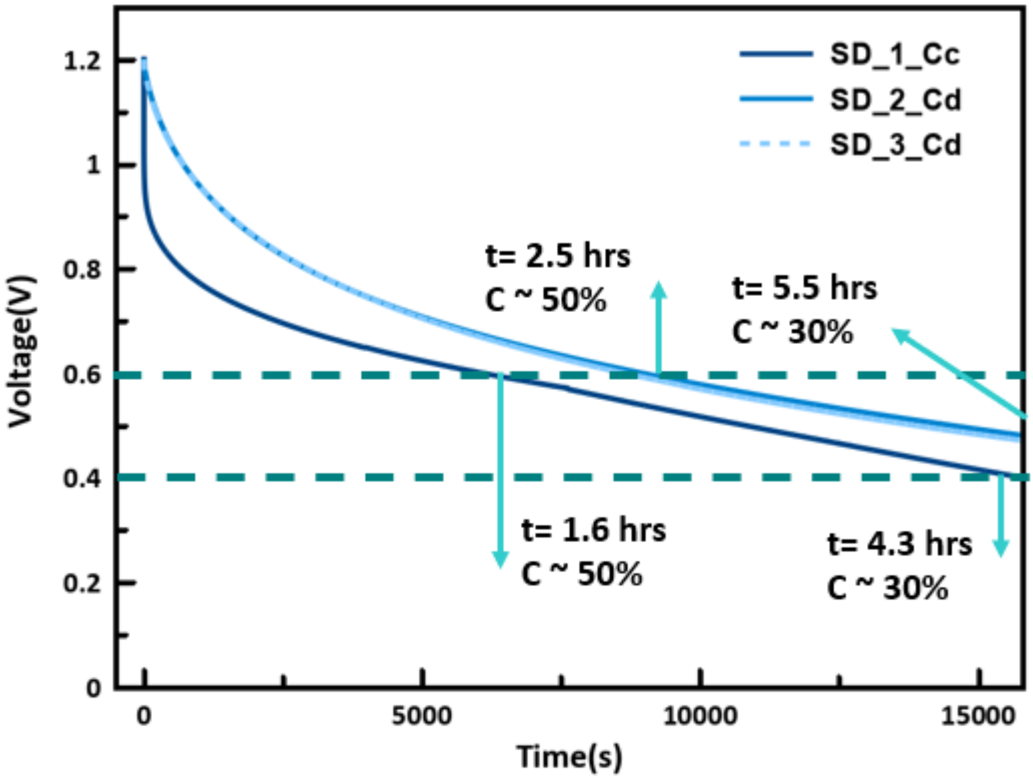

Figure S4. Self-Discharge of two P(ETE-S) root-based supercapacitors in series that have been charged to 1.2V via the OPV when only the OPV has been disconnected (Circuit connected Cc) or when all circuitry has been disconnected (Circuit disconnected Cd).

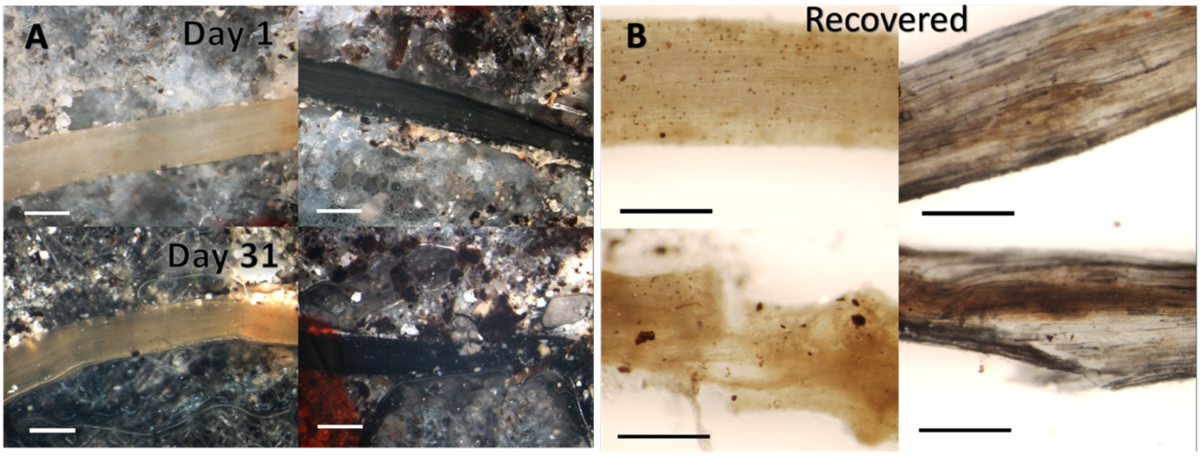

Figure S5. (A). Micrographs of non-functionalized roots and p(ETE-S) roots in soil during degradation assay at Day 1 and Day 31. (B) Recovered roots after Day 31 showing loss of integrity. Scalebar 500um.

Current (μA)	Avg. Capacitance (mF)	Avg. ESR (KΩ)
10	4.7821	1.415
20	4.4583	1.437
30	4.2523	1.395
40	4.0858	1.385
50	3.8591	1.36

Table S1. Average values of capacitance and ESR of four different supercapacitors for different charging currents
